# Supplementary material for: Positioning Accuracy in Holographic Optical Traps
Source: Micromachines (Basel). 2021 May 15;12(5):559. doi: 10.3390/mi12050559 (PMC8156812; doi:10.3390/mi12050559)
Supplement: Supplementary file 1 [file micromachines-12-00559-s001.zip › micromachines-1178146-supplementary.pdf]

micromachines

MDPI

Supplementary Material: Positioning Accuracy in Holographic Optical Traps

Frederic Català-Castro [1,2] and Estela Martín-Badosa [1,2,*]

1 Optical Trapping Lab, Grup de Biofotònica (BiOPT), Departament de Física Aplicada, Universitat de Barcelona, 08028 Barcelona, Spain; frederic.catala@icfo.eu
2 Institut de Nanociència i Nanotecnologia (IN2UB), 08028 Barcelona, Spain
* Correspondence: estela.martinb@ub.edu

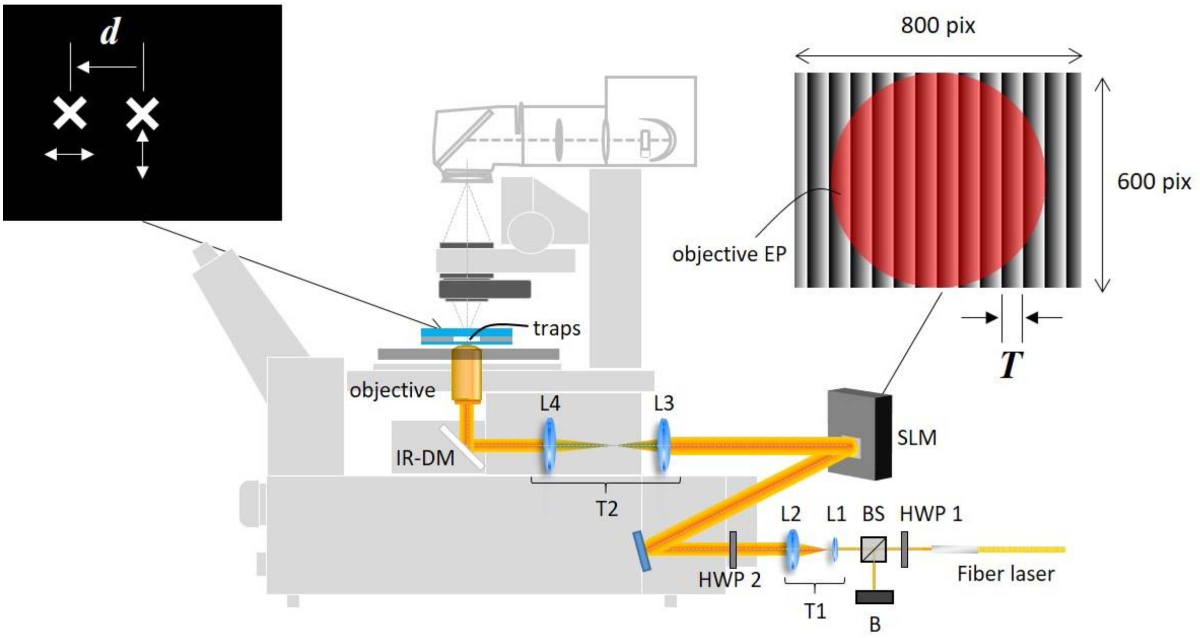

Figure 1. Sketch of our holographic optical tweezers (HOT) set-up. Briefly, our SLM is conjugated at the entrance pupil of an NA = 1.2 trapping objective, so the optical field at the trapping plane is related to the SLM through an optical Fourier Transform. HWP: half-wave plate; B: beam block; BS: polarizing beam splitter; L: lens; T: telescope; IR-DM: infrared short-pass dichroic mirror. See manuscript for details.

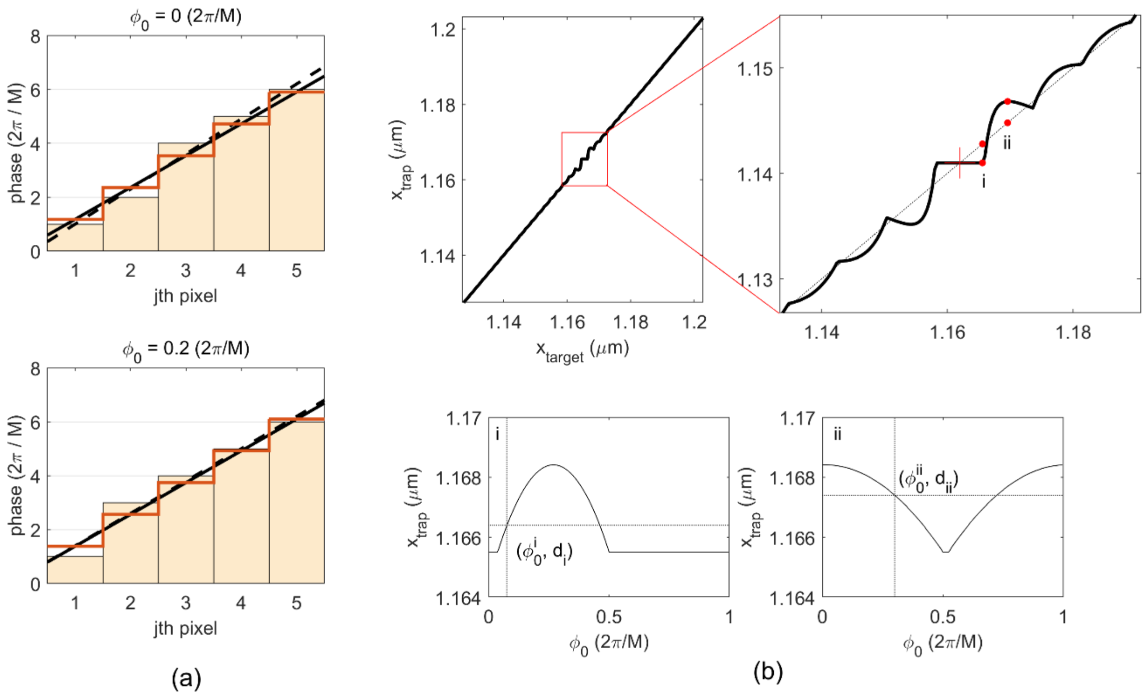

Figure 2. Phase quantization effect on trap positioning. (**a**) Hologram with reduced number of pixels (N = 5). The black line is the ideal, continuous linear phase profile, $\phi^{ideal}(x)$, whereas the dashed line is the average phase profile obtained from the linear fit $\phi_j = ax_j + b$. The orange staircase represents the ideal discrete phase values, ϕ_j^{ideal}, and the bar plot corresponds to the nearest phase values, ϕ_j^{actual}, that the hologram takes after phase quantization. Top – slope difference for a non-optimized case. Bottom – after adding an appropriate phase value, ϕ_0, the phase profile approaches the target slope thanks to a change in pixel 2. (**b**) Trap positioning simulated around $d_{288}^{(1)}$. Insets i and ii show the variation in positioning around d_i and d_{ii} (horizontal, dashed lines) after adding ϕ_0. Phase offsets, ϕ_0^i and ϕ_0^{ii}, optimizing for d_i and d_{ii}, respectively, are indicated.
